# Simultaneous Object and Category Score Estimation in Joint Correspondence Analysis

**DOI:** 10.1017/psy.2025.12

**Published:** 2025-04-07

**Authors:** Naomichi Makino

**Affiliations:** Research Division, National Center for University Entrance Examinations, Tokyo, Japan

**Keywords:** direct method, factor analysis, joint correspondence analysis, joint plot, simultaneous estimation

## Abstract

Joint correspondence analysis (JCA) is a statistical method for obtaining a low-dimensional representation of multivariate categorical data. It was developed as an alternative to multiple correspondence analysis (MCA). Typically, the solution is visualized through a map that projects the data onto a reduced space. A joint map, which shows both object and category scores in the same space, helps users explore inter- and intra-relationships in objects and categories. However, unlike MCA, current JCA estimation methods do not allow the joint representation of objects and categories on the map, which limits the interpretability of JCA results. To overcome this limitation, we propose a simultaneous object and category score estimation method for JCA while addressing the underestimated variance problem that is inherent in MCA. In the proposed method, JCA parameters are estimated by minimizing the discrepancy between the observed categorical data and the JCA data model, rather than relying on the JCA covariance model used in existing estimation methods. Previous research has shown that JCA is comparable to exploratory factor analysis. We also address the factor-analytic interpretation of JCA solutions in addition to geometric interpretation. Two real data analysis examples are also presented to demonstrate the geometric and factor-analytic interpretations of the JCA solutions.

## 1 Introduction

In the social and behavioral sciences, researchers frequently collect the data on how objects are featured by nominal categorical variables. For instance, in social surveys using questionnaires, respondents are asked to select one category from the available options for each item. Multiple correspondence analysis (MCA) is a widely used technique to obtain a low-dimensional graphical representation of multivariate categorical data while preserving crucial information (Beh & Lombardo, 2014; Greenacre, 1984; Le Roux & Rouanet, 2010). The MCA solution provides a joint plot that simultaneously visualizes both objects and categories in the reduced space. This joint graphical display allows users to explore inter- and intra-relationships among objects and categories, which is one of the main advantages of MCA (Hoffman & Franke, 1986).

While MCA is the reasonable method for reducing the dimensionality of multivariate categorical data, it has a considerable drawback: the percentage of variance explained by the solution is underestimated in MCA (Greenacre, 1988, 1994). To address this issue, Greenacre (1988) proposed joint correspondence analysis (JCA) as an alternative to MCA. Subsequent research has explored additional JCA estimation procedures beyond Greenacre’s original method (Boik, 1996; Tateneni & Browne, 2000).

JCA addresses the variance underestimation problem, but the existing JCA methods primarily focus on exploring the relationships between categories in the reduced space with less attention given to object score estimation, except for Boik (1996). In that study, a two-stage estimation procedure for obtaining the object score was proposed. First, the category scores are estimated, and then the object scores are derived based on the estimated category scores. However, the object score estimation step is optional and often omitted in real data analyses. Additionally, no justification was provided in Boik (1996) for jointly displaying the object and category scores. As discussed in Hoffman and Franke (1986), the joint plot is valuable for examining the relationships among the objects and categories, but this representation is not supported by current JCA methods. This limitation is a disadvantage of JCA compared to MCA.

The purpose of this article is to propose an alternative JCA formulation that allows the users to represent the object and category scores jointly in the same low-dimensional space while addressing the underestimated variance problem that is inherent to MCA. In the proposed method, object and category scores can be simultaneously estimated, in contrast to the two-stage estimation procedure employed in the previous study.

As noted in Greenacre (1991); Boik (1996) and van de Velden (2000), JCA solutions can be interpreted in a manner similar to exploratory factor analysis (EFA). From the EFA perspective, the simultaneous JCA estimation method proposed in this article is based on the direct method used in EFA, while the JCA estimation methods introduced by Greenacre (1988) and Boik (1996) are analogous to the MINRES method (Harman & Jones, 1966). The direct method was presented by Professor Henk A. L. Kiers at the University of Groningen in 2001 (Sočan, 2003, p. 17), and de Leeuw (2004) independently developed an equivalent method. Then, this method was revisited in some papers (Adachi, 2019; Stegeman, 2016; Unkel & Trendafilov, 2010). Previous studies have primarily focused on the geometric interpretation of JCA solutions, with the factor-analytic perspective being largely overlooked, despite some mention of the EFA-JCA connection. In this study, we also address the factor-analytic interpretation of JCA solutions in addition to the geometric interpretation.

The organization of this article is as follows. Section 2 provides a brief overview of existing MCA and JCA formulations. Section 3 introduces the proposed alternative JCA formulation and its estimation algorithm. Section 4 explores both the geometric and factor-analytic interpretations of the JCA solution. Because JCA has rotational indeterminacy similar to simple and MCA, Section 5 examines rotation techniques to enhance the interpretability of the JCA solution, drawing on literature from the simple and MCA (Adachi, 2004; Lorenzo-Seva et al., 2009; Makino, 2022; van de Velden, 2000; van de Velden & Kiers, 2003, 2005). Section 6 presents two real data examples to illustrate the geometric and factor-analytic interpretations of JCA solutions. Finally, Section 7 offers our concluding remarks.

## 2 Existing formulations of multiple and JCA

### 2.1 MCA

Assume that we obtain an *n*-objects 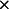 *p*-nominal variables data matrix. This categorical data matrix can be transformed into an *n*-objects 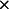 *K*-categories super-indicator matrix 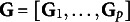 , where 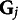 is an *n*-objects 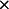 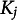 -categories indicator matrix for *j*th variable and 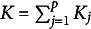 is the total number of categories. Additionally, let 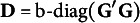 be a *K*-categories 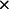 *K*-categories block diagonal matrix whose *j*th block diagonal part is expressed as 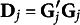 . Also, 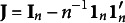 denotes the 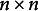 centering matrix.

The MCA data model can be described as follows (Adachi, 2020; Beh & Lombardo, 2014): 
(2.1)

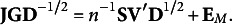

Here, **S** is an *n*-objects 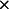 *c*-components object score matrix, 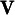 is a *K*-categories 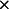 *c*-components category score matrix and 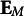 is an *n*-objects 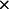 *K*-categories matrix that contains error. The goal of MCA is to find a low-dimensional representation of the observed categorical data while preserving as much of the original variation as possible. The MCA solutions are obtained by minimizing the following loss function 
(2.2)

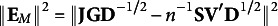

over **S** and **V**.

Let us denote the rank of 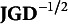 as *r* and the singular value decomposition (SVD) of this matrix as 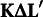 . Here, 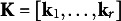 , and 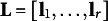 are the left and right singular vector matrices, respectively, satisfying 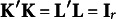 , and 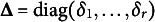 is a diagonal matrix with diagonal elements representing the singular values arranged in descending order. Minimizing the loss function in equation (2.2) can be achieved by 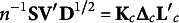 , where 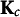 and 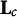 are the matrices consisting of the first *c* columns of **K** and **L** respectively, and 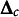 is a diagonal matrix containing the first *c* largest singular values on its diagonal (Eckart & Young, 1936; ten Berge, 1993). Typically, a normalization condition 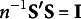 is imposed on **S** for identification purposes. The optimal solutions in MCA can be described as follows: 
(2.3)

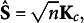



(2.4)

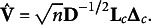

Note that 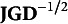 leads to the column-centered left singular vector matrix: the object scores in MCA can be regarded as the standardized scores.

### 2.2 JCA

Boik (1996) defined the JCA data model as 
(2.5)

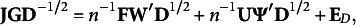

where 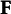 is a common object score matrix ( 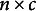 ), 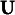 is a unique object score matrix ( 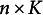 ), 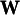 is a common category score matrix ( 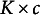 ), 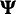 is a unique category score matrix ( 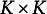 ), 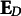 is an error matrix in the data model ( 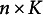 ), respectively. It is assumed that these parameters satisfy the following constraints: 
(2.6)

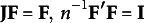



(2.7)

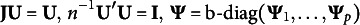



(2.8)

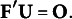

Here, 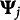 expresses a unique category score matrix of *j*th variable and 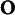 expresses a matrix of zeros. The JCA parameter estimation in Boik (1996) and Greenacre (1988) is based on the covariance model rather than the data model; the corresponding covariance model is described as 
(2.9)

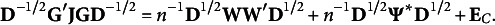

Here, 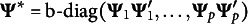 and 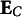 expresses an error matrix in the JCA covariance model ( 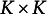 ). The JCA solutions are obtained by minimizing the discrepancy between off-diagonal block parts of 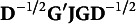 and the corresponding model part, as defined in Greenacre (1988): 
(2.10)

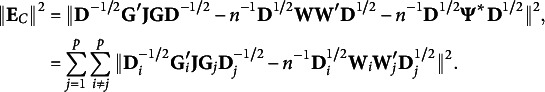

Here, 
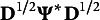
 are assumed to be dummy parameters that are used to fit the diagonal blocks of 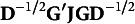 . Greenacre (1988) proposed a JCA estimation algorithm that alternates two minimization steps until a convergence criterion is satisfied: (a) minimize with respect to 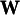 while keeping 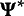 fixed and (b) minimize with respect to 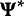 while keeping 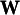 fixed. In step (a), 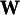 is updated using the eigenvalue decomposition (EVD) of 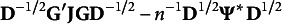 . In step (b), 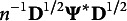 is updated using 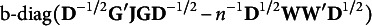 . This algorithm is analogous to the MINRES algorithm introduced by Harman and Jones (1966) in factor analysis.

Greenacre (1991) noted that JCA solutions can be interpreted similar to factor analysis: the diagonal block of 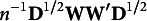 is regarded as the variance explained by the common factor, while 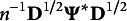 is the variance explained by the unique factor. For these interpretations to be vaild, the unique variance part should be positive semi-definite (psd). However, in some cases, this psd assumption is violated, leading to what is known as an improper solution or a Heywood case in the context of factor analysis. Boik (1996) developed a modified JCA estimation algorithm to ensure that the unique variance part remains psd, thereby avoiding the Heywood case.

## 3 Simultaneous object and category score estimation in JCA

In JCA, the object and category scores can be obtained simultaneously by fitting the data model, rather than the covariance model, to the observed categorical data. This approach is achieved through the proposed JCA loss function 
(3.1)

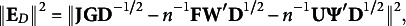

is minimized over **F**, **U**, **W**, and 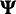 , subject to constraints (2.6), (2.7), and (2.8). As detailed in the following subsections, the proposed JCA estimation algorithm primarily involves two iterative minimization steps: (i) updating the common and unique object scores and (ii) updating the common and unique category scores. These steps are alternated until a pre-specified convergence criterion is satisfied. This algorithm is analogous to the direct method or matrix decomposition factor analysis in factor analysis (Adachi, 2019; de Leeuw, 2004; Sočan, 2003; Stegeman, 2016; Unkel & Trendafilov, 2010), whereas the existing JCA algorithm corresponds to the MINRES method.

### 3.1 Update object scores

By denoting 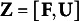 and 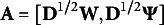 , (3.1) is rewritten as 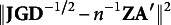 . This function can be expanded as 
(3.2)

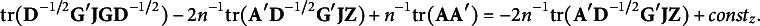

Here, 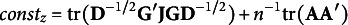 is a constant that does not depend on the update of object scores. In this expression, the constraints (2.6), (2.7), and (2.8) related to object scores are replaced by 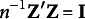 and 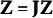 .

Equation (3.2) indicates that minimizing over **Z** is equivalent to maximizing 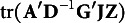 over **Z** for a given **A**. As demonstrated by ten Berge (1993), the following inequality holds for this trace function: 
(3.3)

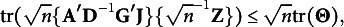

where the SVD of 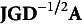 is denoted as 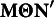 . The upper bound is attained for 
(3.4)

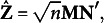

under the constraint 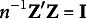 , implying that **Z** can be updated by (3.4). 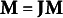 holds in this SVD, and thus the updated object scores satisfy the column-centered constraints. This update can be regarded as solving the Procrustes problem; the first *c* columns of 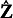 correspond to 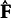 , while the remaining columns of 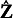 corresponds to 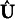 (de Leeuw, 2004; Unkel & Trendafilov, 2010).

### 3.2 Update category scores

First, the minimization over **W**, given **F**, **U**, and 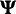 is considered. The proposed loss function can be rewritten as 
(3.5)

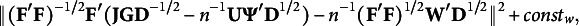

where 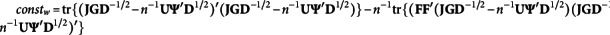 , which is irrelevant to the update of **W**. It can be seen from (3.5) that the minimum value for **W** is attained when 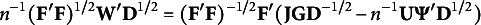 . Consequently, the common category score matrix can be updated by 
(3.6)

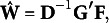

which is equivalent to solving an unconstrained matrix regression problem (ten Berge, 1993). Equation (3.6) shows that common category scores are the centroids of the common object scores that share the same category, indicating that the JCA solution has the same property as the MCA solution.

Next, we address the minimization over 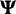 , given **F**, **U**, and **W**. This minimization is achieved by solving the unconstrained regression problem for each 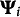 , as shown by the following reformulation of the loss function: 
(3.7)

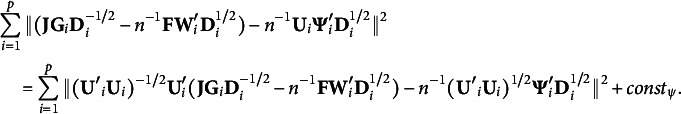

Here, 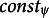 is irrelevant for updating 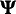 and can be expressed as 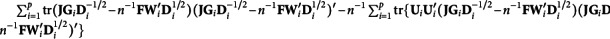 . Therefore, the unique category score matrix can be updated by 
(3.8)

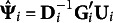

for each *i* ( 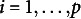 ). Accordingly, the update of 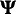 can be described as 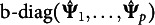 .

### 3.3 Whole algorithm and its properties

According to the discussion in previous subsections, the proposed alternating least square algorithm is summarized as follows:

1. Initialize the object and category scores. The initial object score matrices 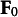 and 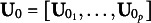 are randomly generated in such a way that the constraints (2.6), (2.7), and (2.8) are satisfied. Then, initial 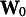 and 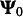 are obtained by 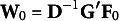 and 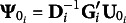 for each *i*, respectively.
2. Update the object score matrices **F** and **U**, given the category score matrices as described by equation (3.4).
3. Update the category score matrices **W** and 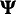 , given the object score matrices.
  1. Update the common category score matrix using equation (3.6).
  2. Update the unique category score matrices for each variable using equation (3.8).
4. Repeat Steps 2 and 3 until the pre-specified convergence criterion is met.

The ALS algorithm of the direct method monotonically decreases the loss function and converges to a local minimum (Unkel & Trendafilov, 2010). To avoid accepting a local minimum, we run the algorithm 100 times with different initial values. Among the resulting multiple solutions, the one with the lowest loss function value is chosen as the optimal solution. For example, the initial object score matrices can be obtained using the SVD of a random matrix: generate a column-centered random matrix ( 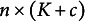 ) with the full column rank, and then the left singular vectors of the random matrix can be used as the initial object score matrices. Step 4 defines convergence as the difference in loss function values between the current and previous rounds being less than 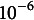 .

The proposed method has two considerable properties: it avoids producing the Heywood case and the object scores are uniquely undetermined. In the proposed method, 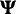 is parameterized as the coefficient matrix for **U**, ensuring that the unique variances part 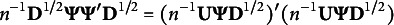 satisfies the psd constraint. This prevents the occurrence of the Heywood case never occurs, unlike the existing JCA estimation procedures where 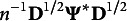 is directly estimated without the psd constraint.

Recall that the update of **Z** is based on the SVD of 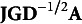 . It is known that the rank of 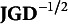 is 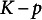 , which implies that the rank of 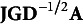 is at most 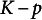 . Let us denote the rank of 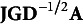 as *r* ( 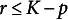 ). The SVD of 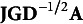 is described as 
(3.9)

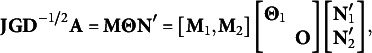

where the size of matrices 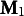 , 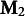 , 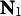 , 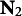 , and 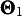 are 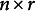 , 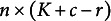 , 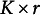 , 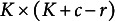 and 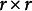 , respectively. This expression leads to 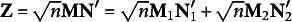 . We find that 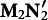 and thus **Z** are uniquely undetermined, indicating that JCA has a factor indeterminacy property in the same manner as factor analysis.

## 4 Interpretation of the JCA solution

### 4.1 Geometric interpretation

The optimal category score matrix in MCA is expressed as 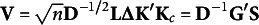 . Substituting 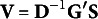 into (2.2), we have 
(4.1)

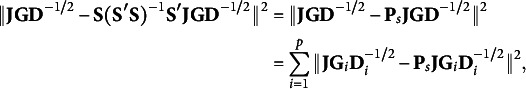

where 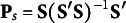 is an orthogonal projection matrix. This indicates that MCA aims to find a subspace spanned by **S** that preserves as much of the variation in the observed categorical data as possible. The joint map interpretation is validated because **S** and **V** are considered the coordinates of objects and categories in this subspace (Greenacre, 1984).

As demonstrated in the previous section, the optimal common category score matrix in JCA is given by 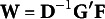 . Substituting 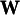 into (3.1), we have 
(4.2)

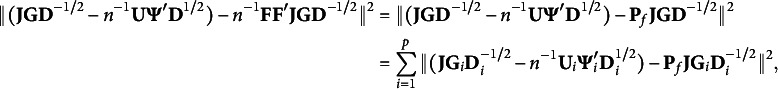

where 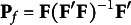 is an orthogonal projection matrix that projects onto the subspace spanned by **F**. As demonstrated below, 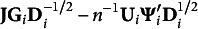 is mutually orthogonal to 
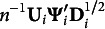
 for each variable ( 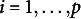 ): 
(4.3)

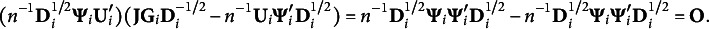

This result shows that the unique part removes variances that are irrelevant to estimating the common part from the observed categorical data.

Equations (4.1) and (4.2) show that MCA and JCA aims to find subspaces spanned by the common components that preserve essential information inherent in the original categorical data. The MCA and JCA solutions can be interpreted as coordinates in the joint map by projecting the observed data onto these subspaces. While the MCA and JCA are quite similar, the unique part distinguishes them. JCA can provide a low-dimensional representation of the observed categorical data retaining the variation after discarding irrelevant variances to obtain the common part.

### 4.2 Factor-analytic interpretation

JCA is closely related to factor analysis as discussed in Greenacre (1988, 1991); Boik (1996), and van de Velden (2000), but its solution is typically interpreted only geometrically. Here, we explore the factor-analytic interpretation of the JCA solution.

In factor analysis, common factor loadings are usually interpreted. Let us denote that 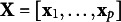 is a standardized quantitative data matrix ( 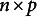 ) and 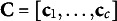 is a common factor score matrix ( 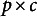 ) that satisfies 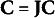 and 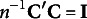 . A common factor loading matrix 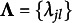 can be defined as 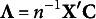 , which contains the cosines between the column vectors of **X** and 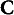 : 
(4.4)

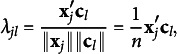

which illustrates the relationship between latent factors and observed variables. The common factor loadings reflect the extent to which the common factors are associated with the observed variables.

In JCA, 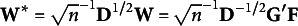 corresponds to the common loading matrix because the elements of 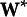 are equivalent to the cosines between the column vector of **G** and **F**: 
(4.5)

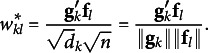

Here, 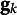 is the *k*th column vector of **G**, 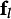 is the *l*th column vector of **F**, and 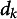 is the *k*th diagonal element of **D**. Thus, 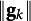 represents the square root of the number of objects responding to the corresponding category, which equals the square root of the *k*th diagonal element of **D**. Consequently, we can understand how each category relates to each common factor by examining the common loading matrix 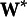 in JCA.

In the geometric interpretation, solutions are typically represented as points in a two-dimensional plot. However, there are cases where a three-dimensional or higher-dimensional approximation is necessary to accurately capture the variation in the observed categorical data (Lorenzo-Seva et al., 2009; van de Velden & Kiers, 2005). In such situations, the factor-analytic interpretation provides a valuable alternative when the geometric interpretation becomes challenging.

### 4.3 Explained variance (EV) by the common factor

EV by the common factor is a measure of the quality of the *c*-dimensional approximation. However, the EVs in MCA and JCA are defined differently depending on the unique part.

The MCA loss function can be rewritten as 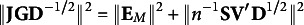 , which shows that the total variation in the categorical data is decomposed into the common and error components. This decomposition leads to the following equation: 
(4.6)

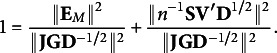

The second term in (4.6) indicates the extent to which the *c*-dimensional solutions accounts for the total variation in the standardized categorical data. This is referred to as the proportion of EVs (PEV). It has been noted that in MCA, the PEV may be underestimated because 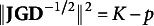 , which is the denominator of the PEV, can be artificially inflated (Greenacre, 1988, 1994).

In the proposed JCA formulation, the EV can be defined similarly to MCA. Equation (3.1) simplifies to 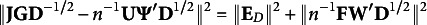 . This implies that the PEV in JCA is expressed as 
(4.7)

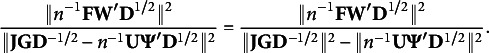

Equation (4.7) shows how well the common factors account for the variation in the original data after excluding the unique variances from the observed data. Let us recall that the JCA loss function is described as 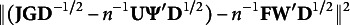 , implying that 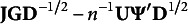 is approximated by 
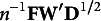
 . The PEV in JCA can also be regarded as an index of how well the common parts explain the target to be approximated. Compared to the PEV in MCA, the unique part reduces inflation of the denominator term, thereby providing a more accurate assessment of the quality of the approximation quality in JCA.

Next, we consider the decomposition of the variances explained by the common part: 
(4.8)

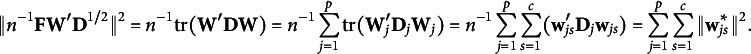

Here, we denote a submatrix in the JCA loading matrix as 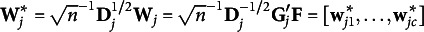 ( 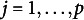 ). 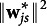 represents the contribution of the *s*th common factor to the variance of *j*th variable and can be rewritten as follows: 
(4.9)

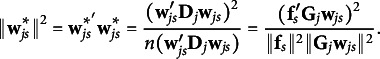

In this context, the squared loading is equivalent to the squared correlation between 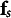 and 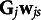 , which corresponds to the discrimination measure (Gifi, 1990). Squared loadings serve as an index that assesses how the *j*th variable relates to the *s*th common factor in JCA. In the factor-analytic interpretation, squared loadings are crucial for understanding the solutions alongside ordinary loadings.

Equation (4.8) can be restated as 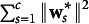 , where 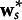 is the *s*th column vector of 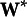 . The contribution rate of the *s*th common factor to the variance in the original data after eliminating the unique factor variances is defined as 
(4.10)

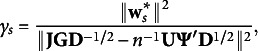

and then 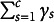 is equal to (4.7).

## 5 Rotational indeterminacy in JCA

Denote **T** as an arbitrary nonsingular matrix ( 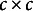 ). The JCA solutions in the proposed formulation can be transformed by **T** without affecting the fitness of the loss function: 
(5.1)

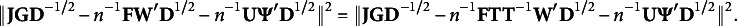

To address this transformational indeterminacy, we can impose restrictions on **T** to achieve orthogonal or oblique rotation. Specifically, **T** can be constrained to be an orthogonal matrix for orthogonal rotation, or 
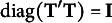
 for oblique rotation. This allows the solution to be rotated in an orthogonal or oblique manner to enhance interpretability. Various rotation criteria have been proposed to facilitate this interpretablity (Browne, 2001; Sass & Schmitt, 2010).

It is a common practice to rotate the solution in the context of factor analysis and principal component analysis. Similarly, orthogonal and oblique rotations have been applied to simple and MCA solutions to improve the interpretability. These previous studies falls into two categories: the first category includes studies that employ rotation to help the factor-analytic interpretation of the solution (Adachi, 2004; Makino, 2022), while the second category comprises studies that utilize rotation to facilitate the geometric interpretation of the solution (Lorenzo-Seva et al., 2009; van de Velden, 2000; van de Velden & Kiers, 2003, 2005). The insights gained from rotating solutions in the simple and MCA can be extended to both factor-analytic and geometric interpretations of the proposed JCA solutions.

In factor-analytic interpretation, rotation is used to achieve a more interpretable loading matrix. As previously discussed, 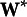 is the common loading matrix in JCA. In this context, the rotation in JCA aims to simplify the loading matrix by finding an optimal rotation matrix. The orthogonal and oblique rotation criteria established in EFA can similarly be applied to determine the optimal rotation matrix for the JCA solution.

When rotating solutions with geometric interpretation, it is important to consider the differences between symmetric and asymmetric plots (Lorenzo-Seva et al., 2009; van de Velden, 2000; van de Velden & Kiers, 2003, 2005). In symmetric plots, object and category coordinates are treated equally; both sets of coordinates are rotated simultaneously to have the simple structure. In contrast, asymmetric plots assign different roles to the coordinates. Here, the position of the points in one coordinate, known as the principal coordinate, is determined by calculating the weighted average of the points in the other coordinate, referred to as the standard coordinate. In the asymmetric plot scenario, the principal coordinate matrix functions similarly to the loading matrix in EFA. The goal of rotation in this context is to find a rotation matrix that enhances the interpretability of the principal coordinate matrix. For the proposed JCA solutions, which aligns with the asymmetric plot, **W** represents the principal coordinate matrix. Thus, rotation in JCA with geometric interpretation aims to identify a rotation matrix that simplifies the interpretation of the common category score matrix. Both orthogonal and oblique rotation criteria from previous studies can be applied to determine the optimal rotation matrix.

While the JCA solutions can be rotated, it is not always necessary, especially in the context of geometric interpretation. Rotation in geometric interpretation is primarily designed to aid the interpretation of high-dimensional solutions that are challenging to represent in two-dimensions (Lorenzo-Seva et al., 2009; van de Velden, 2000; van de Velden & Kiers, 2003, 2005). Previous studies have indicated that rotation may not enhance geometric interpretation of the low-dimensional solutions. The JCA solutions without rotation are preferable in geometric interpretation if the low-dimensional approximation effectively captures the variation in the observed data. On the other hand, for higher-dimensional JCA solutions, orthogonal or oblique rotations are more suitable for improving interpretability.

In factor-analytic interpretation, both orthogonal and oblique rotations can be applied to two- or multidimensional solutions, similar to the methods used in EFA. However, these rotations impact the interpretation of squared loadings differently. In the case of oblique rotation, inter-correlation between the common factors must be excluded to accurately interpret squared loadings. On the other hand, the orthogonal rotation does not affect the interpretation of squared loadings because it ensures that the rotated factors remains uncorrelated. Therefore, the orthogonal rotation may be preferable interpreting both ordinary and squared loadings.

## 6 Real data examples

This section provides two real data examples to illustrate the proposed JCA procedure. The first example highlights the geometric interpretation, while the second example focuses on the factor-analytic interpretation. In this section, objects can be rewritten as respondents for the sake of interpretation.

### 6.1 Psychological student data

In the first example, we use psychological student data (Adachi, 2004) originally presented by Adachi (2000) in Japanese and later translated into English by Adachi (2004). The data comprises responses from thirty students majoring in social or clinical psychology at a Japanese university. The dataset includes answers to five questions:

- Which do you major in? (2 categories): Social psychology (S) or Clinical psychology (C).
- Which method do you like for investigating psychology? (3 categories): Experiment (EXP), Survey (SURV), or Interview (INTs).
- Which interests you besides psychology? (4 categories): Sociology (SOC), Anthropology (ANTH), Medicine (MED) or Literature (LIT).
- Which do you think mainly determines human behavior? (2 categories): Inner mind (I-Mind), Outer environment (O-Env).
- Which subject do you like? (5 categories): Mathematics (MATH), Natural Sciences (NSCI), Social Sciences (SOCS), English (ENG), or Japanese (JPN).

In this example, our goal is to compare the joint display of JCA and MCA solutions. We used two-dimensional solutions, as done by Adachi (2004). The PEV for both MCA and JCA solutions is detailed in Table 1. The MCA solution accounted for the 33.9% of the variation in the psychological student data. Consistent with Greenacre (1988, 1994), the total PEV for MCA was also found to be under-assessed. In contrast, the JCA solution explained a substantial portion of the data variation after eliminating the unique variances, with the total PEV of 90.5%.

**Table 1:** PEV (%) of the JCA and MCA solutions in the psychological student dataset

	Dimension1	Dimension2	Total
JCA	51.3	39.2	90.5
MCA	18.4	15.5	33.9

**Figure 1:**
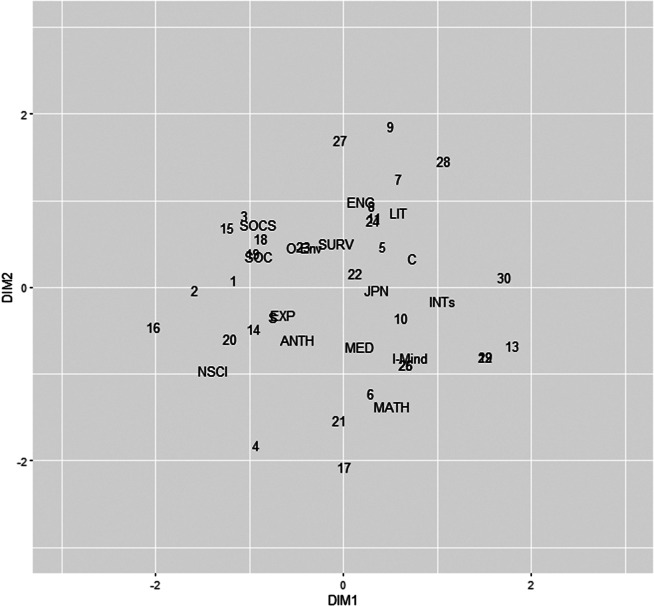
Two-dimensional joint plot of the MCA solution.

**Figure 2:**
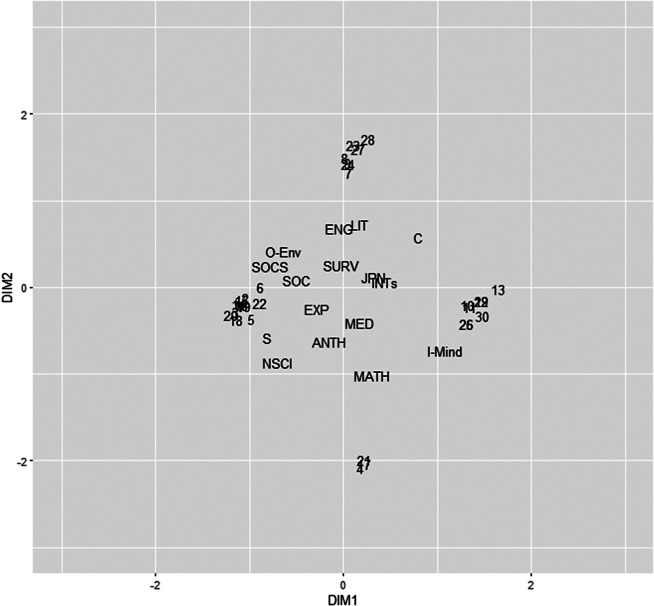
Two-dimensional joint plot of the JCA solution.

Figures 1 and 2 show the unrotated joint plots of respondents and category scores derived from the MCA and JCA procedures, respectively: **S** and **V** are jointly plotted in Figure 1, and **F** and **W** are jointly plotted in Figure 2. The category scores from both MCA and JCA exhibit similar patterns. However, the respondent scores reveal a notable distinction: the JCA respondent scores are grouped into four clusters, whereas the MCA respondent scores are more dispersed. The clustered solution makes it easier to interpret the patterns of the respondents than the dispersed solution because we can easily interpret the factor scores simply by focusing on the clusters. Based on the PEV and visualization, it can be concluded that the JCA solution provides more insightful results compared to the MCA solution.

### 6.2 Taste data

In this illustration, our goal is to demonstrate the interpretation of higher-dimensional solution in JCA, with a focus on the factor-analytic perspective. A comparison with MCA results is not addressed here. We use taste data obtained from Le Roux and Rouanet (2010) for this demonstration. This dataset includes responses from 1,215 individuals on the following four nominal items:

- Preferred TV program (eight categories): TV-News, TV-Comedy, TV-Police, TV-Nature, TV-Sport, TV-Films, TV-Drama, and TV-Soap operas.
- Preferred film (eight categories): Action, Comedy, Costume drama, Documentary, Horror, Musical, Romance, and SciFi.
- Preferred type of art (seven categories): Performance, Landscape, Renaissance, Still life, Portrait, Modern art, and Impressionism.
- Preferred place to eat out (six categories): Fish & Chips, Pub, Indian restaurant, Italian restaurant, French restaurant, and Steak house.

In this example, we used a three-dimensional JCA solution, consistent with the analysis presented in Le Roux and Rouanet (2010). Table 2 presents the PEV for the JCA solution. The estimated common factors accounted for 96.2% of the variances in the taste data after excluding unique variances, indicating that the three-dimensional solution adequately captured the variation.

**Table 2:** PEV (%) of the JCA solution in the taste dataset

	Dimension1	Dimension2	Dimension3	Total
JCA	43.7	22.2	30.3	96.2

For interpretation, we used both ordinary and squared loadings. To facilitate the interpretation of the ordinary loading matrix, we applied varimax rotation (Kaiser, 1958), an orthogonal rotation method, which does not affect the interpretation of squared loadings. Tables 3 and 4 show the unrotated and varimax-rotated ordinary and squared loadings, respectively. These tables show that each category and variable primarily loads on a single factor, with minor cross-loadings. The rotated solutions more closely approach the perfect simple structure compared to the unrotated solutions.

**Table 3:** Unrotated and Varimax-rotated JCA loadings for the taste data

		Unrotated			Varimax		
		Dimension1	Dimension2	Dimension3	Dimension1	Dimension2	Dimension3
Item1	TV-Comedy	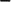 0.081	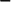 0.137	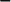 0.234	0.029	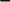 0.149	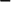 0.239
	TV-Drama	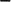 0.110	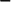 0.058	0.257	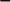 0.178	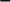 0.079	0.209
	TV-Films	0.099	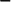 0.089	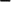 0.220	0.181	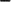 0.068	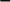 0.169
	TV-Nature	0.314	0.001	0.030	0.280	0.061	0.132
	TV-News	0.284	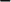 0.012	0.119	0.225	0.042	0.206
	TV-Police	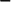 0.030	0.061	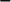 0.023	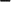 0.031	0.055	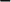 0.035
	TV-Soap operas	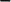 0.733	0.163	0.245	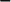 0.790	0.020	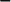 0.022
	TV-Sport	0.348	0.047	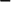 0.277	0.406	0.113	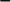 0.149
Item2	Action	0.084	0.031	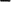 0.105	0.107	0.047	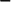 0.073
	Comedy	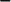 0.195	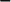 0.060	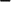 0.248	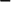 0.086	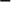 0.094	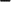 0.294
	CostumeDrama	0.170	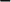 0.056	0.452	0.016	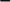 0.025	0.485
	Documentary	0.197	0.046	0.166	0.118	0.082	0.218
	Horror	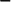 0.092	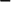 0.105	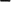 0.224	0.009	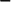 0.119	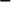 0.235
	Musical	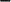 0.027	0.149	0.104	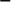 0.086	0.141	0.080
	Romance	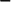 0.267	0.048	0.016	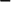 0.260	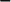 0.003	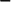 0.076
	SciFi	0.099	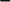 0.054	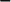 0.048	0.117	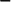 0.034	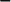 0.009
Item3	Impressionism	0.046	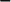 0.299	0.110	0.060	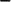 0.286	0.137
	Landscape	0.095	0.305	0.015	0.028	0.318	0.027
	Modern Art	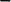 0.116	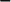 0.255	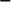 0.108	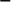 0.025	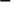 0.272	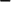 0.124
	Performance Art	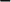 0.035	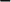 0.045	0.013	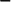 0.029	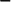 0.051	0.004
	Portrait	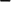 0.150	0.065	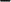 0.093	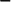 0.119	0.036	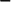 0.141
	Renaissance Art	0.086	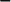 0.128	0.117	0.063	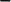 0.110	0.146
	Still Life	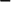 0.042	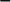 0.110	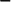 0.057	0.000	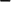 0.116	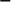 0.061
Item4	Fish&Chips	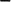 0.040	0.154	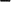 0.023	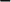 0.058	0.144	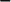 0.044
	French restaurant	0.120	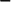 0.140	0.183	0.075	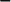 0.115	0.220
	Indian restaurant	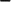 0.078	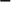 0.130	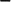 0.153	0.003	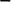 0.142	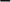 0.162
	Italian restaurant	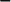 0.002	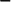 0.214	0.029	0.027	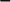 0.210	0.040
	Pub	0.035	0.224	0.052	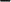 0.026	0.227	0.047
	Steak house	0.025	0.188	0.017	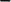 0.017	0.189	0.013

**Table 4:** Unrotated and Varimax-rotated JCA squared loadings for the taste data

	Unrotated			Varimax		
	Dimension1	Dimension2	Dimension3	Dimension1	Dimension2	Dimension3
TV	0.868	0.063	0.322	0.984	0.055	0.214
Film	0.203	0.048	0.367	0.122	0.054	0.443
Art	0.058	0.283	0.050	0.024	0.286	0.080
Eat	0.024	0.192	0.061	0.011	0.186	0.081

Dimension 1 shows a positive association with TV-Sports, TV-Nature and TV-News, while negatively relating to TV-Soap operas and TV-Drama. The former categories can be grouped as “Non-fictional,” and the latter as “Fictional,” indicating that this dimension reflects a preference between Non-fictional and Fictional TV. Dimension 2 is positively associated with Landscape, Fish&Chips, Pub, and Steak house, but negatively associated with Impressionism, Modern art, Italian restaurant, and Indian restaurant. This dimension appears to contrast “popular” tastes with “sophisticated” ones. Dimension 3 has positive associations with CostumeDrama, Documentary, TV-Drama and TV-News, and negative associations with Comedy, Horror, TV-Comedy and TV-Films. The positively associated categories represent a “matter of fact” taste group, while the negatively associated categories reflect an “affective content” taste group in film production.

## 7 Concluding remarks

We developed a simultaneous object and category score estimation method for JCA, which is based on the JCA data model rather than the JCA covariance model. Additionally, we provided a rationale for the joint graphical representation of the objects and categories. While the proposed JCA formulation is similar to the MCA formulation, the unique part is a key term for addressing the underestimation problem identified in equation (4.6). Consequently, users can explore the inter- and intra-relationships among the objects and categories while effectively mitigating the underestimation problem seen in MCA, as illustrated by the psychological student data example.

It is important to note that while the common factor scores exhibit a clustered structure in the real data example, this does not imply that JCA will always produce such interpretable scores. In the direct method, the factor score matrix **Z** = [**F**, **U**] cannot be uniquely determined, with **Z** being the sum of determinate and undetermined matrices. This implies that **F** used in Figure 2 is one of the optimal **F**’s. Procrustes analysis was used to estimate **F** in the real data example, but other methods are adaptable if a desirable matrix can be reasonably selected from a set of the undermined matrices that satisfy the conditions. Uno et al. (2019) proposed a procedure called clustered common factor exploration (CCFE), designed to estimate the common factor score matrix that is as well classified as possible in the framework of the direct method. CCFE allows for the selection of the undetermined part to achieve a clustered structure in the factor estimation step. CCFE is applicable to the proposed JCA estimation procedure by replacing Step 2 with CCFE because the same estimation procedures are used in the direct and the proposed methods. CCFE may be a desirable option when observing large numbers of objects.

The JCA solutions are commonly interpreted through graphical maps, but they can also be analyzed similarly to EFA, where the relationships between variables and common factors are examined using loadings defined by (4.5). Although two-dimensional solutions are typically employed for geometric interpretation, there are instances where three-dimensional or higher-dimensional approximation is more suitable, making visualization more complex. In such cases, factor-analytic interpretation serves as an effective alternative, as illustrated by the analysis of the taste data example.

Rotation was an overlooked topic in JCA, but this is crucial for interpreting the obtained solutions. This article discussed how and when to rotate the JCA solutions, emphasizing the need to consider the differences between geometric and factor-analytic interpretations. While this article focuses on rotation to achieve the interpretable loadings, another type of rotation is discussed in the context of PCAMIX, which encompasses MCA as a special case (Kiers, 1991). In PCAMIX, rotation is applied to make squared loadings more interpretable (Adachi, 2004; Chavent et al., 2012; Kiers, 1991). Future research could explore how to transform JCA solutions to achieve a simpler structure.

In the estimation method proposed by Boik (1996) and Greenacre (1988), unique variances can become negative if the psd constraint is not imposed. In contrast, the proposed method consistently produces non-negative unique variances without requiring additional constraints. Non-negative unique variances are crucial for JCA because they help mitigate the artificial inflation of observed categorical data variances when calculating the PEV. Specifically, the denominator of the PEV in JCA, given by 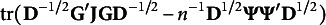 , is lower than the denominator of the PEV in MCA, which is 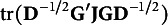 . For this comparison to be valid, unique variances must be non-negative; otherwise, negative unique variances could exacerbate inflation and fail to address the underestimation problem.

Recent investigations have explored the mathematical properties of the direct method (Adachi & Trendafilov, 2018, 2019; Adachi, 2022; Stegeman, 2016). Further research into these properties with respect to JCA, particularly focusing on the mathematical distinctions between MCA and JCA, would be valuable in future studies.

## Data Availability

The data that support the findings of this study are openly available. Psychological student data is available within Adachi (2004), while taste data can be downloaded from the webpage of Dr. Le Roux, who is an author of Le Roux and Rouanet (2010). A program used in the JCA estimation is provided from https://osf.io/wc4vs/?view_only=f089be0b11454a538125c6843cf5acc1.
